# Delay Analysis of GTS Bridging between IEEE 802.15.4 and IEEE 802.11 Networks for Healthcare Applications

**DOI:** 10.1155/2009/987046

**Published:** 2008-12-02

**Authors:** Jelena Mišić, Xuemin (Sherman) Shen

**Affiliations:** ^1^Department of Computer Science, University of Manitoba, Winnipeg, MB, Canada R3T 2N2; ^2^Department of Electrical and Computer Engineering, University of Waterloo, Waterloo, ON, Canada N2L 3G1

## Abstract

We consider interconnection of IEEE 802.15.4 beacon-enabled network cluster with IEEE 802.11b network. This scenario is important in healthcare applications where IEEE 802.15.4 nodes comprise patient's body area network (BAN) and are involved in sensing some health-related data. BAN nodes have very short communication range in order to avoid harming patient's health and save energy. Sensed data needs to be transmitted to an access point in the ward room using wireless technology with higher transmission range and rate such as IEEE 802.11b. We model the interconnected network where IEEE 802.15.4-based BAN operates in guaranteed time slot (GTS) mode, and IEEE 802.11b part of the bridge conveys GTS superframe to the 802.11b access point. We then analyze the network delays. Performance analysis is performed using EKG traffic from continuous telemetry, and we discuss the delays of communication due the increasing number of patients.

## 1. Introduction

**Figure 1:**
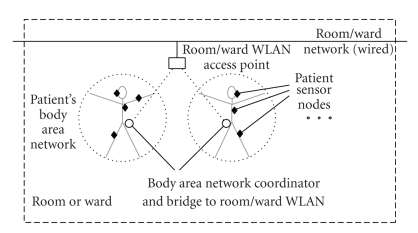
Networking structure of the ward room with BANs, bridges, and ward LAN.

Wireless sensor networks in healthcare applications require small lightweight devices with sensing, computational, and communication features to be unobtrusively placed on patient's body. They need to communicate results of sensing of healthcare data periodically over very short range to the devices which can be carried by the patient or mounted on patient's bed. We refer to the wireless sensor network on patient's body as body area network (BAN). Low power, that is, short communication range of wireless sensors is needed for health, interference, and security reasons. Devices which collect the results of measurements need to provide some limited data processing and aggregation, add security/privacy functions, and communicate aggregated data to the LAN access point in patient's ward room as shown in Figure 1. We refer to this device as bridge.

In this paper, we consider interconnection of IEEE 802.15.4 beacon-enabled network cluster with IEEE 802.11b network. The IEEE 802.15.4 nodes comprise patient's body area network (BAN) and are involved in sensing some health-related data which will be transmitted to the access point in the ward room using wireless technology such as IEEE 802.11b. It is clear that the network performance depends on the characteristics of the interconnection device. Therefore, we model the interconnected network where IEEE 802.15.4-based BAN operates in guaranteed time slot (GTS) mode, and IEEE 802.11b part of the bridge conveys GTS superframe to the 802.11b access point. We then analyze the impact of important parameters such as acceptable load ranges and the delays of communication due the increasing number of patients. Since real-time operation of the bridges is necessary for many measurements in hospitals besides intensive care units (ICUs), we will study communications through the bridge for simple case of EKG continuous telemetry.

The remainder of the paper is organized as follows. We review the networking aspect of healthcare wireless sensor networks with the emphasis on continuous electrocardiogram telemetry in Section 2. In Section 3, we review the properties of 802.15.4 beacon-enabled MAC and IEEE 802.11b MAC related to the operation of bridge. In Section 4, we present the concepts of bridge design between IEEE 802.15.4 BAN and IEEE 802.11b ward LAN. Analytical model of bridge where BAN operates in GTS mode is presented in Section 5. In Section 6, we present performance results for interconnected clusters under various modes of bridge access. Finally, Section 7 concludes the paper.

## 2. Wireless Sensor Networks for Healthcare

In today's hospitals, there is an urgent need for timely monitoring the health status of many patients, especially those with respiratory and cardiac problems. The need for continuous fetal heart rate monitoring as well as monitoring for movements of patients suffered from stroke or Parkinson's disease is also high. Although these applications require different kinds of sensors to measure levels of oxygen in blood, heart rate, or motion of body parts, there is unified need for sensors to be unobtrusively attached to the patient's body and for measured data to be transmitted in a reliable and secure way in order to be recorded on monitoring devices in real time. Most of the health variables are periodic and have to be periodically sampled and digitized. The sampling period has to be at least twice as large as the highest frequency of the healthcare variables.

Recently, several medical telemetry applications have been prototyped so far and moved to production phase such as pulse oximeters, EKG devices, and motion analysis systems [1]. Wireless transmission is currently implemented using Bluetooth IEEE 802.15.1 and IEEE 802.11b technologies although IEEE 802.15.4 emerges as most suitable for medical applications due to its low-power and low-bandwidth requirements. Some initial work in this area has been reported in [1–4]. However, current reports on wireless healthcare products are focused on reliable hardware and software designs of sensor modules while the wireless transmission has been considered in testing phase and for single device only. There is an area of research involving coordination and real-time transmission of large number of healthcare measurements, which has not attracted sufficient attention. In this paper, we will address the following problems.

1. Interconnection of low-power IEEE 802.15.4 motes (which are convenient for attachment on patient's body) with IEEE 802.11b network which has larger bandwidth and larger transmission range. We will design bridge between IEEE 802.15.4 wireless communication interface(s) residing at patient's body and IEEE 802.11b residing at bedside or carried in patient's pocket. More specifically, we will use TDMA feature of IEEE 802.15.4 called guaranteed time slots to fill it with digitized samples and pass it to the interface of IEEE 802.11b for further transmission to the hospital room's access point.
2. Analysis of delay of real-time health measurement data incurred by transmission technologies. We will use measurement data for electrocardiogram and analyze traffic when the number of patients increases.

Security is another important issue in wireless healthcare sensor networks. In this paper, we will also address data integrity issue by assuming that there is a shared secret key between 802.15.4 sensor mote and bridge device. Secret key will be used to provide message authentication code in each 802.15.4 superframe (packet) using HMAC function [5, 6].

### 2.1. EKG Measurement

Electrocardiogram (ECG or EKG) is a surface measurement of the electrical potential generated by the electrical activity, which controls pumping action of cardiac muscle fibers. These electrical impulses generate voltage, which further generates current flow in the torso and potential differences on the skin. Standard EKG monitoring involves short-term (≤30 seconds) monitoring of heart pulses using 12 skin electrodes (called leads) placed at designated locations on the patient's body including chest, arms, and legs. Each pair of leads measures voltage which gives one aspect of the heart's activity. An EKG picture produced by 12 leads allows diagnosis of wide range of heart problems. However, this measurement is short-term and requires wired connection between the patient and electrocardiograph.

In many cases, however, it is necessary to have continuous and tetherless measurements of heart rate. For such application, only three leads placed at patient's upper and lower chest can trace a wide range of cardiac arrhythmias. One node of these three collects signals, amplifies the signal difference, samples the amplified analog signal, and digitizes it. Standard clinical EKG application has the bandwidth of 0.05 Hz to 100 Hz. For pacemaker detection, upper frequency can be up to 1 kHz. There are many design issues out of scope of this work related to noise suppression and filtering frequencies from power line and respiration. In this paper, we assume that upper frequency of the EKG signal is 100 Hz. EKG signal is sampled with 200 Hz, and each sample is digitized with 12 bits [3]. Therefore, basic bandwidth of EKG signal in standard continuous telemetry is only 2400 bps.

## 3. Basic Properties of IEEE Std 802.15.4 and IEEE 802.11b MACs

### 3.1. Basic Properties of IEEE Std 802.15.4 MAC

**Figure 2:**
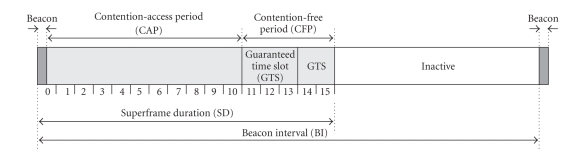
The composition of the superframe under IEEE Std 802.15.4 (adopted from [7]).

In beacon-enabled networks, the personal area network (PAN) coordinator divides its channel time into superframes [7]. Each superframe begins with the transmission of a network beacon, followed by an active portion and an optional inactive portion, as shown in Figure 2. The coordinator interacts with its PAN during the active portion of the superframe, and may enter a low-power mode during the inactive portion. Raw data rate in industrial, scientific, and medical (ISM) band is 250 Kbps. Basic time unit in the standard is backoff period which contains 10 bytes. Duration of active and inactive parts of the superframe is regulated with MAC parameters *S* *O* = 0,…, 14 which is known as *macSuperframeOrder* and *B* *O* = 0,…, 14, also called *macBeaconOrder*. Active superframe part is divided into 16 slots. Each slot consists of 3·2^*SO*^ backoff periods, which gives the shortest active superframe duration *aBaseSuperframeDuration* of 48 backoff periods when *S* *O* = 0. Duration of an active superframe part is denoted as *SD* = *aBaseSuperframeDuration *·2^*SO*^ (superframe duration). The time interval between successive beacons is equal to *BI* = *aBaseSuperframeDuration *∗ 2^*BO*^. The duration of the inactive period of the superframe can be determined as *I* = *aBaseSuperframeDuration *∗ (2^*BO*^ − 2^*SO*^). Period between the beacons is equal to the active superframe duration only if there is no inactive period in the cluster time, and, otherwise, it is larger than active superframe part, that is, *B* *O* ≥ *S* *O*.

An active superframe part consists of contention part and TDMA, that is, guaranteed time slot (GTS) part. GTS bandwidth must be requested by the node using the MAC command frame. Coordinator allocates the GTS bandwidth in multiples of slots. One slot contains 3 ∗ 2^*SO*^ backoff periods. Data transfer from a node to PAN coordinator can be done in GTS slots or using slotted CSMA-CA access described below. Slotted CSMA-CA algorithm consists of backoff activity, two clear channel assessments (CCAs), packet transmission, and optionally receipt of the acknowledgment. Backoff value is uniformly chosen in the range (0, *w*_15_ − 1) which is called contention window. During backoff countdown node does not listen to the medium, and checks the activity on the medium only twice when backoff count is finished. By default, the node can have *m*_15_ + 1 = 5 transmission attempts with backoff window sizes *W*_15,0_ = 8, *W*_15,1_ = 16, *W*_15,2_ = 32, *W*_15,3_ = 32, and *W*_15,4_ = 32. To avoid confusion, we will use subscript 15 to label MAC parameters which have their counterparts in the IEEE 802.11b standard.

### 3.2. Basic Properties of IEEE 802.11b Needed for Bridging

IEEE 802.11 has much more sophisticated CSMA-CA scheme at the MAC layer. In this protocol (opposite to IEEE 802.15.4), a station having a packet to transmit must initially listen to the channel to check if another station is transmitting. If there is no transmission in distributed interframe space (DIFS) time interval, the transmission can proceed. If medium is busy, the station has to wait until current transmission has finished. Then, station will wait for DIFS time period and then generate a random backoff time before transmitting its frame. This backoff time is uniformly chosen in the range (0, *w*_11_ − 1). Backoff counter will be decremented after each *slot time* given that transmission medium is free, otherwise, its value will be frozen until medium becomes free again for DIFS time units (slot time is derived from the propagation delay time to switch from receiving to transmitting mode and time to pass the information about the physical channel state to MAC layer. It actually corresponds to backoff period from IEEE 802.15.4). Station will transmit when its backoff counter reaches zero value. When the packet is received, receiver replies with acknowledgment (ACK) packet after short interframe space (SIFS) time interval. Whenever packet collision occurs, acknowledgment will not be received within SIFS + ACK time, and transmission has to be reattempted with doubled contention window. If starting window size is *w*_11_ = *W*_11,min_ after *m*_11_ retransmissions, its maximal value will become *W*_11,max_ = 2^*m*_11_^ *W*_11,min_ (in order to distinguish between similar variables in two standards, we use subscript 11). In our model, we assume that if packet experiences more than *m*_11_ collisions, last backoff stage will be entered for every subsequent retransmission until frame is successfully transmitted. In order to limit packet collision time, and guard against hidden terminal problem, the standard allows small reservation packets request to send (RTS) and clear to send (CTS) sent using CSMA-CA. After transmission of RTS packet, receiver replies with CTS after short interframe space(SIFS) time. Due to sensitivity of healthcare applications, we will assume that RTS/CTS scheme is used to protect packets with measurement data of health variables.

The IEEE 802.11b standard is mostly deployed in current implementations of healthcare wireless sensor networks [1]. The IEEE 802.11b has higher-speed physical layer than original IEEE 802.11 and allows transmissions at 1, 2, 5.5, and 11 Mbps, while IEEE 802.11 supports transmission at 2 Mbps. However, physical layer header is transmitted at 1 Mbps, MAC layer header and payload can be transmitted at 1, 2, 5.5, or 11 Mbps while control frames RTS, CTS, and ACK are transmitted at 1 or 2 Mbps. It is also worth noting that since IEEE 802.11b adapters transmit at a constant power, distances covered with transmission speeds of 1, 2, 5.5, and 11 Mbps are 120, 90, 70, and 30 m, respectively [8].

Starting with seminal work in [9], performance of IEEE 802.11 has been extensively studied first for saturated case and later for unsaturated case [10–13]. Standard extension IEEE 802.11e enhances CSMA-CA access by introducing different interframe spaces and different backoff window ranges for different traffic classes. This scheme has been modeled using similar approach (although more complex) as basic 802.11 scheme in [14–16]. However, given the fact that all BANs have the same priority and short packet sizes with sensing information, we believe that IEEE 802.11b can serve the purpose and that deployment of IEEE 802.11e at this point may not be necessary.

## 4. GTS Bridge Design

**Figure 3:**
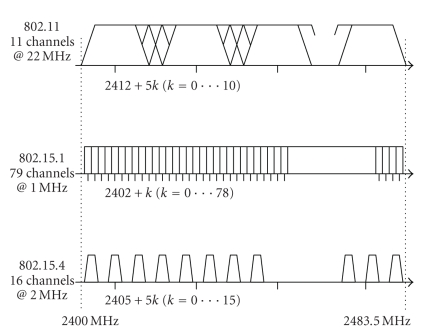
Used spectra of wireless LAN and PAN technologies in ISM band.

Bridge consists of IEEE 802.15.4 PAN coordinator and ordinary IEEE 802.11b interface. These two components are interconnected through a buffer which is filled by the PAN coordinator and emptied by IEEE 802.11b packet transmission facility. IEEE 802.15.4 PAN coordinator interface and IEEE 802.11b interface have their wireless transmit/receive antennas. Both networks operate in ISM band as shown in Figure 3, and there is a need to coordinate operation of bridge's interfaces either in TDMA or FDMA manner. From Figure 2 and discussion in Section 3.1, we observe that it is possible to achieve TDMA coordination between the interfaces using the fact that WLAN bridge interface can operate during silent BAN periods. However, in the presence of multiple BANs within WLAN coverage, this approach requires synchronization of BAN beacons (we assume that interference among BANs is avoided by separation in space or by allocating separate channels as shown in Figure 3). Also bandwidth allocation through *S* *O* and *B* *O* parameters must be achieved such that bridged traffic from all BANs can be delivered during (common) inactive superframe part.

It is much more convenient if operation of BAN and WLAN is separated in frequency domain because BAN beacons do not need to be synchronized and more bandwidth is allocated to the bridge. BAN channels should be chosen in such way that they do not overlap with ward WLAN channel. From Figure 3, we see that each WLAN channel overlaps with four BAN channels. Therefore, for each ward WLAN channel, 12 out of 16 BAN channels should be used in order to avoid interference.

Duration of beacon interval *B* *I* is tuned according to period of sensed health variable. Duration of active period *S* *D* is chosen in order to

1. achieve data transmission with high success probability in the case of CSMA-CA MAC,
2. in case of GTS transmission of sensed data, GTS bandwidth has to match necessary size of a group of GTS packets and acknowledgment lanes where group size corresponds to the number of IEEE 802.15.4 nodes in BAN. However, some small contention periods must be reserved in the superframe in order to communicate command frames between sensing nodes and PAN coordinator.

Since bandwidth of IEEE 802.11b is much larger than the bandwidth of IEEE 802.15.4 BAN, transmission of one IEEE 802.11b packet will take short time, and rest of active period can be used to exchange some command data between the bridge and access point of the ward room. Between two transmission phases, sensing nodes and bridge are idle and can turnoff their transmitters and receivers. Duration of this sleep time is *B* *I* − *S* *D*. We make an important remark about time scales in IEEE 802.15.4 and IEEE 802.11b. Duration of backoff period in 802.15.4 is 320 microseconds, and with raw data rate of 250 Kbps, one backoff period carries 10 bytes of data. IEEE 802.15.4 slot duration is 3∗2^*SO*^ backoff periods. On the other hand, IEEE 802.11b backoff counter is decremented after slot time which is equal to 20 microseconds. Therefore, time-scale translation is needed between two networks.

We assume that traffic intensity due to sensing of health variables in BAN will be light given that the number of nodes is lower than 16, and that offered load per node is lower than few Kbps. This is well below the rate of 250 Kbps supported by IEEE 802.15.4.

**Figure 4:**
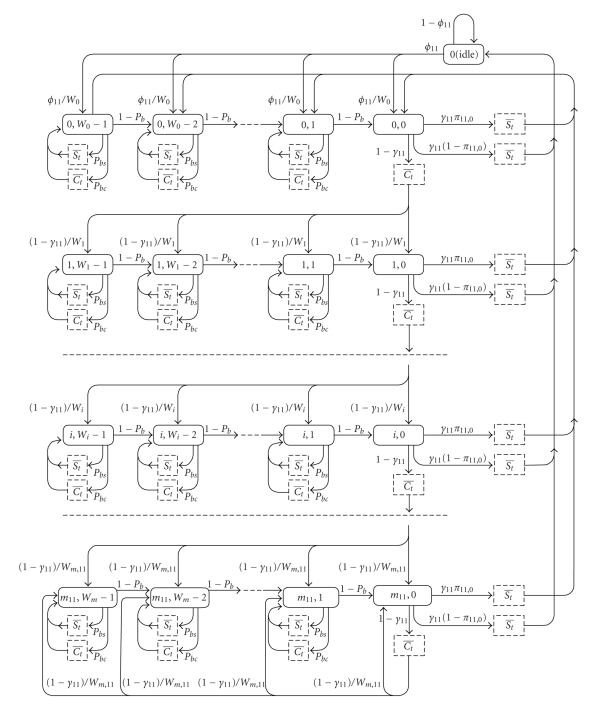
Markov chain for IEEE 802.11b coupled with device's queue.

Bridge's buffer will be served by IEEE 802.11b CSMA-CA MAC for which the Markov chain model is presented in Figure 4. In our modeling, we will assume that data buffers at BAN nodes and at the bridge are infinite. Although, this assumption may appear unrealistic for sensor nodes (and we have always modeled small finite buffers in our work [17]); offered load to the node is low so that node's buffer is empty most of the time. Therefore, both the queuing model with finite and infinite buffers will give similar results. Given the lower computational complexity of the model with infinite buffer, we use it in our analysis although use of finite buffer model is straightforward. Assumption of infinite buffer at the bridge is reasonable since it may contain more complex hardware and software, and offered load per bridge is low-to-moderate, depending on the number of patients in vicinity of access point.

## 5. Analytical Model of GTS Bridging

In general case of GTS, bridge sensors may report different medical variables, such as heart rate, level of oxygen in blood, and temperature. Each sensor is allocated a number of slots in uplink direction to carry uplink sensing data and a slot in downlink direction to carry acknowledgment. We will refer to the number of slots in uplink direction as *packet lane*.

PAN coordinator receives data from stations in separate GTS packet lanes, generates acknowledgments, and passes aggregated packets to IEEE 802.11b buffer.

Assume that each sensor needs to be served with data rate *D*_*s*_ bps. This results in allocation of *d*_*s*_ packet lanes such that

(1) $$D_{s}\;=\;\frac{d_{s}{*3*2}^{SO}*80}{{48*2}^{BO}*0.00032},$$

and that period between the beacons *B* *I* = 48 ∗ 2^*BO*^ ∗ 0.00032 matches the sampling period of health variable. Assuming that there are *n*_15_ < 16 sensors in the BAN, each sensor will have one GTS slot, and the last slot will contain aggregated acknowledgments for all sensors. For larger number of sensors where 15(*k* − 1) < *n*_15_ ≤ 15*k* period between the beacons has to be decreased *k* times, such that each superframe carries readings from at most 15 sensors (and acknowledgments in the last GTS slot).

For simplicity, let *n*_15_ < 16 and the payload of the IEEE 802.15.4 superframe becomes payload of IEEE 802.11b frame consisting of *n*_15_·*d*_*s*_·3·2^*SO*^·10 bytes. IEEE 802.11b frame length in bytes has to be augmented with headers from physical and MAC 802.11b layer which is 50 bytes. Finally, we have to find frame size in 802.11b slots (backoff periods), since each slot carries number of bits *s*_11_ equal to the product of raw data rate and slot duration, and its value is equal to *l*_11_ = (50 + *n*_15_·*d*_*s*_·3·2^*SO*^·10)/*s*_11_.

Duration of RTS, CTS, and ACK frames expressed in slots will be denoted as *rts*, *cts*, and *a* *c* *k*_11_, respectively (we will use subscript 11 to denote IEEE 802.11b whenever potential ambiguity may arise between two standards). Duration of DIFS and SIFS periods in slots will be denoted as *difs* and *sifs*.

Probability generating function (PGF) for the successful packet transmission time is equal to [12]

(2) $$St(z)\;=\;z^{rts+cts+l_{11}+3sifs+difs+{ack}_{11}},$$

with average value $\overset{¯}{St}\;=\;St^{\prime }(1)\;=\;rts\;+\;cts\;+\;l_{11}\;+\;3sifs\;+\;difs\;+\;{ack}_{11}$. In the case of collision of RTS packets activity on medium has PGF:

(3) $$Ct(z)\;=\;z^{rts+cts+sifs+difs},$$

with average value $\overset{¯}{Ct}\;=\;Ct^{\prime }(1)\;=\;rts\;+\;cts\;+\;sifs\;+\;difs.$

Assume that there are *n*_11_ bridges attached to the IEEE 802.11b access point. Each bridge communicates the same kind of sensing traffic towards the access point. Using the assumption from previous work [2–5, 9, 14, 15] that probability of successful transmission is independent of the backoff stage, we will denote it as *γ*_11_ while collision probability is 1 − *γ*_11_. Access probability is also independent of the backoff stage and is denoted as *τ*_11_. Relationship between these two probabilities is

(4) $$\gamma_{11}\;=\;{\left(1-\tau_{11}\right)}^{\left(n_{11}-1\right)}.$$

Probability that medium will be active during the backoff countdown of one station has two components. First one is the probability that station sensed the medium busy due to successful transmission of one among *n*_11_ − 1 stations, and it has the value *p*_*bs*_ = (*n*_11_ − 1)*τ*_11_(1 − *τ*_11_)^(*n*_11_−2)^. The other component is the probability that station senses the medium busy due to collision among some of *n*_11_ − 2 other stations and has the value *p*_*bc*_ = 1 − (1 − *τ*_11_)^(*n*_11_−1)^ − *p*_*bs*_. Their sum is the probability that medium is busy during the backoff countdown, and that backoff counter is frozen *p*_*b*_ = *p*_*bs*_ + *p*_*bc*_ = 1 − *γ*_11_. The PGF for the duration of time between two successive backoff countdowns is represented with the following equation [12]:

(5) $$Hd(z)\;=\;z\gamma_{11}+(p_{bc}Ct(z)\;+\;p_{bs}St(z))Hd(z).$$

At this point, we note that duration of period between two successive decrements of backoff counter is limited to the maximum packet size. After transmission is finished and DIFS period passes any station doing the backoff, countdown has to decrement its backoff counter at least once before packet transmission:

(6) $$B_{11,i}(z)\;=\;\overset{W_{11,i}-1}{\underset{k=0}{\sum }}\frac{1}{W_{11,i}}H_{d}^{k}(z)\;=\;\frac{H_{d}^{W_{11,i}}(z)-1}{W_{11,i}(H_{d}(z)-1)},$$

where *W*_11,*i*_ = 2^*i*^ *W*_11,0_ for *i* ≤ *m*_11_, and *W*_11,*i*_ = 2^*m*_11_^ *W*_11,0_ for *i* > *m*_11_.

Assuming that packet will be retransmitted until valid acknowledgement is received, the PGF for the packet service time becomes

(7) $$\begin{array}{ll} T_{11}(z) & \;=\;\overset{m_{11}+1}{\underset{i=1}{\sum }}\left(\overset{i-1}{\underset{j=0}{\prod }}B_{11,j}(z)\right){\left(1-\gamma_{11}\right)}^{\left(i-1\right)}Ct{\left(z\right)}^{\left(i-1\right)}\gamma_{11}St(z)\\ & \;+\;\left(\overset{m_{11}}{\underset{j=0}{\prod }}B_{11,j}(z)\right)\overset{\infty }{\underset{i=m_{11}+1}{\sum }}{\left(B_{11,m_{11}}(z)\right)}^{\left(i-m_{11}\right)}\\ & \;\times \;{\left(1-\gamma_{11}\right)}^{\left(i-1\right)}Ct{\left(z\right)}^{\left(i-1\right)}\gamma_{11}St(z), \end{array}$$

and its average value is obtained as $\overset{¯}{T_{11}}\;=\;T_{11}^{\prime }(1)$.

### 5.1. Markov Chain Model and Queuing Model for the GTS Bridge’s Output

In the derivations above, we have derived probability of successful transmission and probability of freezing the backoff period using the variable which represented access probability per 802.11b slot *τ*_11_. Access probability, on the other hand, has to be derived using two modeling components. First one is the Markov chain which represents conditional activities within the CSMA-CA process. Second component indicates the probability that bridge will be idle and that it will not perform backoff count and attempt transmission. This happens only when the bridge's packet buffer is empty. In order to find this probability, we must deploy queuing theory and we have to know the arrival process to the bridge's queue, the size of the bridge's queue, and the probability distribution packet service time by which packets depart from the queue. Therefore, Markov chain model and queuing model of the bridge are coupled and have to be modeled and solved simultaneously. We will first solve the Markov chain for CSMA-CA MAC using the variable *π*_11,0_ which represents the probability that bridge's buffer is empty after the packet departure.

Since similar Markov chain models have been solved with detailed steps of setting transition probabilities in the past in [9–12], we will just state the most important steps in derivation of access probability. Markov chain {*s*(*t*), *b*(*t*)} is discrete as transitions are observed at ends of slot times. It is bidimensional (given that *γ*_11_ and *p*_*b*_ are independent of backoff stage) where *s*(*t*) represents backoff stage and *b*(*t*) represents value of backoff counter. Corresponding states of the Markov chain will have the state probabilities *y*_*i*,*j*_, *i* = 0,…, *m*_11_, *j* = 0,…, *W*_11,*i*_ − 1. Since packet sizes in this application are relatively small, we have included the states when transmission of RTS/CTS and data packets is going on. Access probability is equal to *τ*_11_ = ∑_*i* = 0_^*m*_11_^ *y*_*i*,0_.

Probability of idle state when bridge's buffer is empty is equal to

(8) $$P_{\text{idle}}\;=\;\frac{\tau_{11}\gamma_{11}\pi_{11,0}}{\phi_{11}},$$

where *ϕ*_11_ denotes average packet arrival rate of the arrival process to the bridge (note that packet arrival process is not Poisson when packets arrive from IEEE 802.15.4 BAN to the bridge).

By inserting all necessary transition probabilities, we obtain

(9) $$\begin{array}{ll} \tau_{11} & \;=\;(\frac{\gamma_{11}\pi_{11,0}}{\phi_{11}}+\overset{m_{11}}{\underset{j=0}{\sum }}\gamma_{11}{\left(1-\gamma_{11}\right)}^{j}+\;\overset{m_{11}}{\underset{j=0}{\sum }}\frac{(W_{11,j}-1)\gamma_{11}{\left(1-\gamma_{11}\right)}^{j}}{2(1-p_{b})}(p_{bc}\overset{¯}{Ct}+p_{bs}\overset{¯}{St})\\ & \;\;+\;{\frac{(W_{11,m_{11}}-1){\left(1-\gamma_{11}\right)}^{\left(m_{11}+1\right)}}{2(1-p_{b})}(p_{bc}\overset{¯}{Ct}+p_{bs}\overset{¯}{St})+\gamma_{11}\overset{¯}{St}+\left(1-\gamma_{11}\right)\overset{¯}{Ct})}^{-1}. \end{array}$$

### 5.2. Derivation of Probability Distribution of Occupancy of Bridge's Buffer

As we mentioned, we will assume that bridge's buffer has an infinite capacity. Rationale behind that is that bridge device indeed can have much larger memory than sensing device and that utilization of the bridge is expected to be light-to-moderate. Therefore, bridge is not expected to work in the regime close to its stability limit where it can reject packets due to finite buffer.

In GTS-based bridge period between arrivals of IEEE 802.15.4 superframes to the bridge is constant, and bridge can be modeled as D/G/1 queuing system modeled at Markov points of packet departures from the bridge. Let us denote period of arrival of sensing information from all the sensors to bridge as Φ_11_ = 1/*ϕ*_11_. As mentioned earlier, if the number of sensors *n*_15_ < 16, then Φ_11_ = *B* *I*, and if 15(*k* − 1) < *n*_15_ ≤ 15*k*, then Φ_15_ = *k* *B* *I*. Assume that PGF for the bridge's packet service time can be expressed as the series as *T*_11_(*z*) = ∑_*k* = 0_^*∞*^ *t*_11,*k*_ *z* ^*k*^. Probability of *l*, *l* = 0, 1,… arrivals of GTS superframes during packet service time of the bridge has the value

(10) $$a_{11,l}\;=\;\text{Prob}[l\Phi_{11}\leq T_{11}<(l+1)\Phi_{11}]=\overset{(l+1)\Phi_{11}-1}{\underset{j=l\Phi_{11}}{\sum }}t_{11,j}.$$

PGF for the probability distribution of the number of 802.15.4 superframe arrivals during packet service time by the 802.11b interface is *A*_11_(*z*) = ∑_*l* = 0_^*∞*^ *a*_11,*l*_ *z* ^*l*^. An equation which shows number of packets in bridge's buffer left after the departure of the packet has the form

(11) $$\pi_{11,l}\;=\;\pi_{11,0}a_{11,l}\;+\overset{l+1}{\underset{j=1}{\sum }}\pi_{11,j}a_{11,l-j+1}.$$

By multiplying both left-hand and right-hand side of (11) with *z* ^*l*^ and summing over *l* = 0,…, *∞*, we obtain PGF for the number of packets left in the bridge's queue after the departing packet Π_11_(*z*) = ∑_*l* = 0_^*∞*^ *π*_11,*l*_ *z* ^*l*^ as

(12) $$\Pi_{11}(z)\;=\;\frac{A_{11}(z)(1-\rho_{11})(1-z)}{A_{11}(z)-z},$$

where $\rho_{11}\;=\;\phi_{11}\bar{T_{11}}$ presents offered load to the bridge.

### 5.3. Output Process and Throughput

Output process from bridge has the PGF for packet interdeparture times as

(13) $$\Delta_{11}(z)\;=\;(1-\pi_{11,0})T_{11}(z)\;+\;\pi_{11,0}z^{\Phi_{11}}T_{11}(z).$$

Assuming that there are *n*_11_ bridges communicating with access point throughput in 802.11b LAN can be then presented with expression

(14) $$\Theta_{11}\;=\;n_{11}\frac{l_{11}-2}{\bar{\Delta_{11}}},$$

where 2 slots correspond to header information.

### 5.4. Distribution of Packet Waiting Time in Bridge's Buffer

Assuming FIFO service discipline, packet arriving at the bridge has to wait for the currently transmitted packet to depart and for complete service time of all packets already queued. According to renewal theory, remaining service time of the packet has PGF [18]

(15) $$T_{11}^{+}(z)\;=\;\frac{\left(1-T_{11}(z)\right)}{\overset{¯}{T_{11}}(1-z)}.$$

Then, PGF for the waiting time of the packet has the form

(16) $$\begin{array}{ll} & W_{11}(z)\\ & =\;\pi_{11,0}\;+\;\pi_{11,1}T_{11}^{+}(z)\;+\;\pi_{11,2}T_{11}^{+}(z)T_{11}(z)\\ & \;+\;\pi_{11,3}T_{11}^{+}(z){\left(T_{11}(z)\right)}^{2}\cdots \\ & =\;\pi_{11,0}\;+\;T_{11}^{+}(z)(\pi_{11,1}\;+\;\pi_{11,2}T_{11}(z)\;+\;\pi_{11,3}{\left(T_{11}(z)\right)}^{2}+\cdots )\\ & =\;\pi_{11,0}\;+\;T_{11}^{+}(z)\frac{\Pi_{11}(T_{11}(z))\;-\;\pi_{11,0}}{T_{11}(z)}\\ & =\;\pi_{11,0}\left(1\;+\;T_{11}^{+}(z)\frac{1\;-\;A_{11}(T_{11}(z))}{A_{11}(T_{11}(z))-T_{11}(z)}\right). \end{array}$$

Average delay can be obtained by differentiating (16) and applying L'Hospital's rule:

(17) $$\overset{¯}{W_{11}}\;=\;\frac{\phi_{11}T_{11}^{\left(2\right)}}{2(1-\rho_{11})},$$

where *T*_11_^(2)^ denotes second moment of packet service time. This result matches Pollaczek-Khinchin mean value formula [19].

The complete access time which includes waiting time and service time of the target packet is then equal to $\bar{S_{11}}\;=\;\bar{W_{11}}\;+\;\bar{T_{11}}$.

## 6. Performance Evaluation of GTS Bridge in Continuous EKG Telemetry

In this section, we present performance results for GTS bridge between IEEE 802.15.4 and IEEE 802.11b deployed in continuous EKG telemetry. In design of GTS bridge for EKG telemetry, we assume that superframe will contain only three GTS parts. First one is management slot used for control communication between IEEE 802.15.4 mote and the bridge. Second part is used to carry digitized EKG samples, and the third part should contain acknowledgment from bridge to mote. Duration of these parts depends on the duration of superframe and time distance between the beacons. For example, if *S* *O* = 0, then superframe including the beacon frame contains 16 slots with three backoff periods each. Duration of beacon frame is 30 bytes (3 backoff periods) since beacon can carry acknowledgment information for previous superframe. Minimal duration of management slot is three backoff periods (30 bytes). However, 14 slots are then left to carry samples and packet authentication code. We assume that HMAC function adopted is constructed from secure hash algorithm (SHA-1 hash [6, 20]) which is a widely used cryptographic hash function with a message digest output of 160 bits. Therefore, 400 bytes are left in the superframe which covers at least 200 digitized samples, that is, measurement period of 1 second. This confirms that *S* *O* = 0 is a correct choice as long as the superframes are sent with the period less than a second. Choice of the *BO* parameter, that is, the period between the beacons is result of contradicting requirements (Table 2 outlines our design options). First requirement is related to low power consumption and asks that *BO* is chosen to be as large as possible, but still able to carry all the samples generated during beacon interval (preferably close to 1 second). Second requirement is related to the packetization delay and reliability. Low packetization delay requires small amount of data in the packet. Second, both IEEE 802.15.4 and IEEE 802.11b (and even IEEE 802.15 1 Bluetooth if it happens to operate in vicinity) operate in ISM band and cause interference to each other. Interference can corrupt the whole superframe, and, therefore, shorter superframe sizes are preferable.

We consider an ideal wireless channel (without noise and fading). MAC and physical layer parameters are given in Table 3 resulting in a period between the superframes of 0.123 seconds.

**Figure 5:**
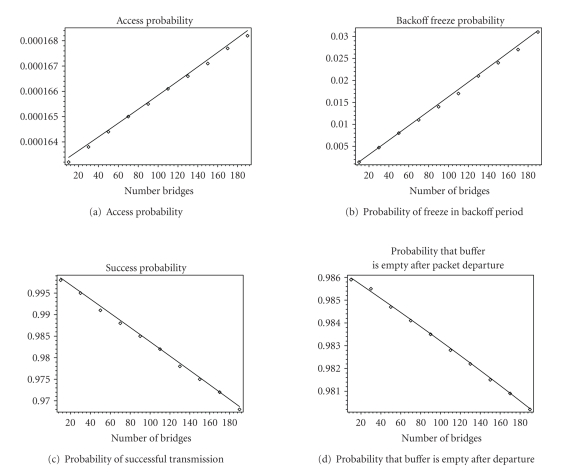
Access probability, probability that backoff count will be frozen, probability of successful transmission, and probability that buffer is empty after departing packet. Analytical results are shown as lines and simulation results are shown as points.

**Figure 6:**
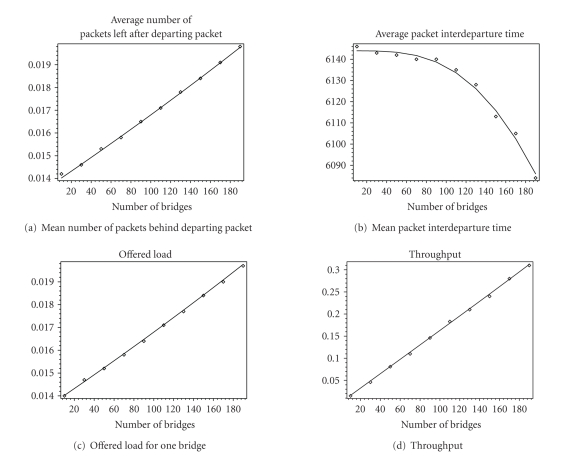
Mean number of packets left in the bridge's queue after departing packet, mean packet interdeparture time, bridge offered load, and throughput. Analytical results are shown as lines, and simulation results are shown as points.

We have numerically solved the overall system of equations under varying number of bridge devices. Analytical processing was done using Maple 11 from Maplesoft. We have also implemented the simulation model using Petri Net simulation engine Artifex [21]. Figures 5 and 6 show values of basic network parameters when the number of bridges is varying between 10 and 190. Delays are shown in numbers of IEEE 802.11b slots (20 microseconds). Although total network load is light, we observe that an increase of the number of devices under constant load per device causes linear increase of access probability, freeze probability, and throughput; while at the same time, transmission success probability, probability that buffer is empty after departure experiences linear decrease. We also observe that analytical (shown as line) and simulation results (shown as points) are close.

**Figure 7:**
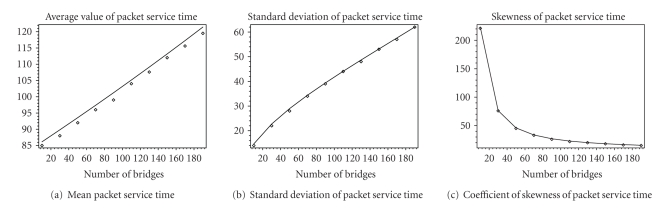
Moments of packet service time. Analytical results are shown as lines and simulation results are shown as points.

**Figure 8:**
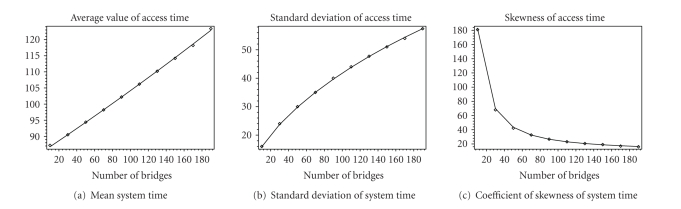
Moments of system (access) time. Analytical results are shown as lines, and simulation results are shown as points.

Figures 7 and 8 show first three moments of the packet service time and packet access time which includes waiting time in the queue and packet service time. We observe that for an increase of the average packet service time of 30%, standard deviation has increased three times. We present coefficient of skewness which is derived as the ratio of the third moment of the probability distribution and third power of standard deviation [22] which indicates symmetry of the probability distribution around the mean value. This coefficient is close to zero for distributions which are symmetric around their mean values. However, calculated values of skewness parameter indicate high level of asymmetry, and we have been motivated to calculate complete probability distributions and discuss them.

**Figure 9:**
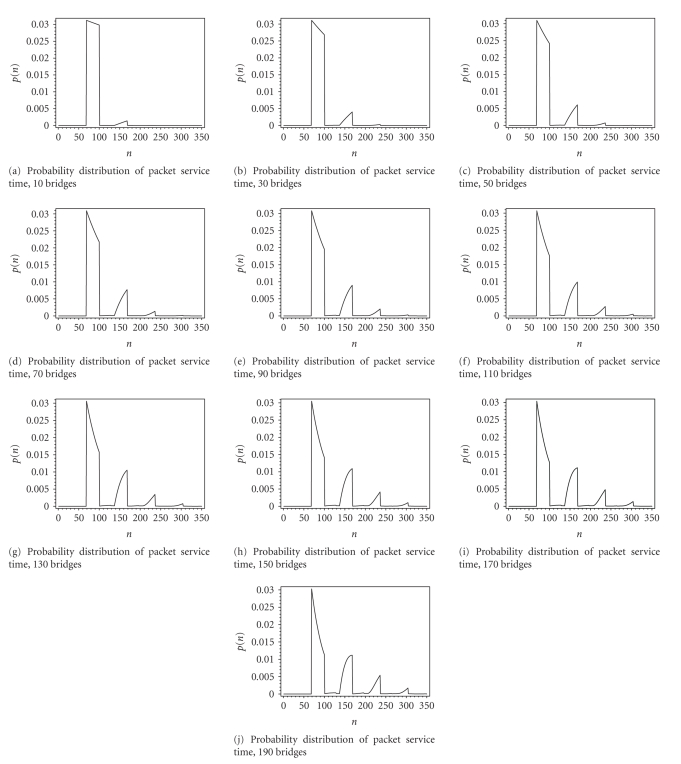
Probability distribution of packet service time.

**Figure 10:**
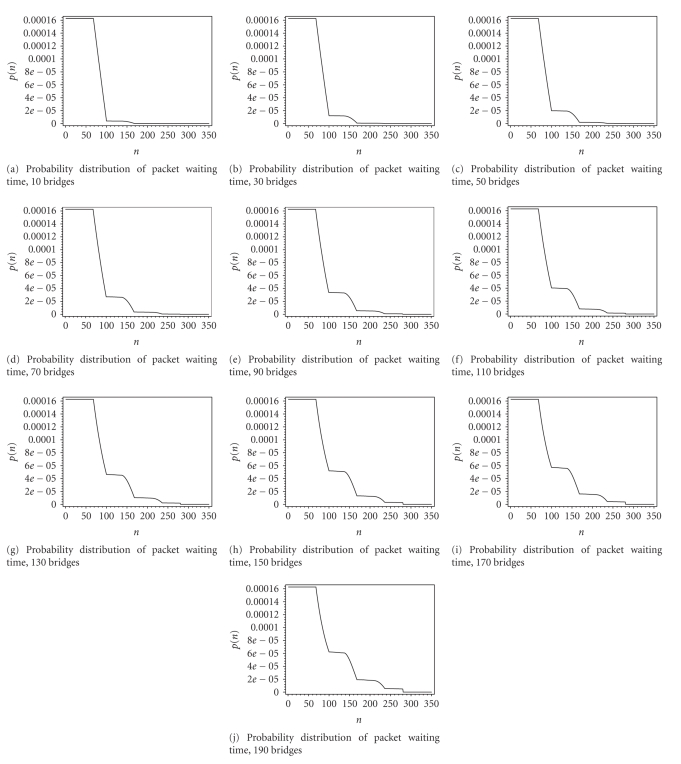
Probability distribution of packet waiting time in bridges' buffer.

Figures 9 and 10 show probability distributions of packet service time and packet waiting time obtained analytically for cases when the number of bridges increases from 10 to 190 in steps of 20. For packet service time, each peak on the graph corresponds to one backoff attempt. We notice that for small number of bridges, almost all packets are served in first backoff attempt. The beginning of the first peak is determined by the time to complete single packet transmission without the backoff count $\bar{St}\;=\;69$ slots. The width of the first peak corresponds to the size of initial backoff window enlarged by freezing. After first backoff, transmission is either successful or collided where collision lasts for $\bar{Ct}\;=\;29$ slots (which corresponds to the distance between the peaks). As the number of bridges increases, the number and intensity of higher-order backoff attempts increases and higher-order peaks become more pronounced. Situation is similar with packet waiting time in the sense that its distribution becomes wider as the number of bridges increases. The first peak of this distribution comes from the residual packet service time (of the packet currently being transmitted) without the collision, and it further widens with increasing number of backoff phases. Flat parts after the first one are due to packet retransmissions after collision.

Both distributions show that packet delay in ward network is random with large variance as the number of bridges increases. This is bad news for real-time payload transmitted in the IEEE 802.11 packets since EKG samples have to be displayed in constant time periods. The results shown are useful to determine the amount of playback buffering for EKG data in order to ensure intelligible display of data.

## 7. Conclusion

In this paper, we have presented the design issues and performance evaluation of the bridge between the BAN implemented using beacon-enabled IEEE 802.15.4 network and IEEE 802.11b wireless LAN. Bridge has been implemented using GTS feature of IEEE 802.15.4. Performance results show that for small offered load and very small packet sizes which carry EKG data (with basic bandwidth of 2400 bps), large number of devices generates very wide probability distribution of the packet access time. Given that EKG data has to be displayed in real time, accurate estimation of access delay is necessary in order to dimension buffering at the receiver. We have shown that probability distributions of packet service time and packet waiting time cannot be characterized using first two moments, instead the whole probability distributions are needed in order to accurately estimate buffering delays at the receiver.

## Figures and Tables

**Table 1:** Comparison of MAC parameters between beacon-enabled IEEE 802.15.4 and IEEE 802.11b.

CSMA-CA parameter	IEEE 802.15.4	IEEE 802.11b
Backoff period size	320 *μ*s	20 *μ*s
Listen to the medium during backoff count	no	yes
Freeze the backoff ctr. when medium is busy	no	yes
Listen to the medium immediately before the trans.	yes	no
Action when medium is sensed busy	New backoff phase	Freeze the backoff ctr.
Typical size of minimum backoff window	8	32
Typical number of backoff phases *m*	5	5
Raw data rate	250 Kbps	1, 2, 5.5 or 11 Mbps
Typical physical + MAC layer header size	48 + 72 bits	192 + 272 bits
RTS and CTS	No in beacon enabled version	Yes

**Table 2:** Tradeoff between packetization delay and number of samples carried in each superframe.

*BO* value	Period between	Number of EKG samples
	beacons	in the superframe
6	0.983s	196
5	0.492s	98
4	0.245s	49
3	0.123s	24

**Table 3:** Parameters used in the analytical modeling.

Number of bridges	20–200
*SO* (802.15.4)	0
*BO* (802.15.4)	3
Raw data rate (802.15.4)	250 Kbps
Superframe size (802.15.4)	480 bytes
MAC and payload data rate for 802.11b	2 Mbps
Payload size of 802.11b packet	10 slots at 2 Mbps
IEEE 802.11 physical + MAC header size	16.4 slots
